# How to survive in the world’s third poplar: Insights from the genome of the highest altitude woody plant, *Hippophae tibetana* (Elaeagnaceae)

**DOI:** 10.3389/fpls.2022.1051587

**Published:** 2022-12-14

**Authors:** Ruoqiu Wang, Bin Wu, Jianbo Jian, Yiwei Tang, Ticao Zhang, Zhiping Song, Wenju Zhang, La Qiong

**Affiliations:** ^1^ Tibet University-Fudan University Joint Laboratory for Biodiversity and Global Change, School of Life Sciences, Fudan University, Shanghai, China; ^2^ Key Laboratory for Biodiversity Science and Ecological Engineering, Institute of Biodiversity Science, School of Life Sciences, Fudan University, Shanghai, China; ^3^ BGI-Shenzhen, Shenzhen, China; ^4^ Research Center for Ecology, College of Science, Tibet University, Lhasa, China

**Keywords:** genome sequencing, *Hippophae tibetana*, whole-genome duplication, high-altitude adaptation, complete genome

## Abstract

*Hippophae tibetana* (Tibetan sea-buckthorn) is one of the highest distributed woody plants in the world (3,000-5,200 meters a.s.l.). It is characterized by adaptation to extreme environment and important economic values. Here, we combined PacBio Hifi platform and Hi-C technology to assemble a 1,452.75 Mb genome encoding 33,367 genes with a Contig N50 of 74.31 Mb, and inferred its sexual chromosome. Two *Hippophae*-specific whole-genome duplication events (18.7-21.2 million years ago, Ma; 28.6-32.4 Ma) and long terminal repeats retroelements (LTR-RTs) amplifications were detected. Comparing with related species at lower altitude, *Ziziphus jujuba* (<1, 700 meters a.s.l.), *H. tibetana* had some significantly rapid evolving genes involved in adaptation to high altitude habitats. However, comparing with *Hippophae rhamnoides* (<3, 700 meters a.s.l.), no rapid evolving genes were found except microtubule and microtubule-based process genes, *H. tibetana* has a larger genome, with extra 2, 503 genes (7.5%) and extra 680.46 Mb transposable elements (TEs) (46.84%). These results suggest that the changes in the copy number and regulatory pattern of genes play a more important role for *H. tibetana* adapting to more extreme and variable environments at higher altitude by more TEs and more genes increasing genome variability and expression plasticity. This suggestion was supported by two findings: nitrogen-fixing genes of *H. tibetana* having more copies, and intact TEs being significantly closer genes than fragmentary TEs. This study provided new insights into the evolution of alpine plants.

## Introduction

The Qinghai-Tibet Plateau (QTP) is one of the most extreme regions in the world, but it is also one of the regions with the highest biodiversity (Myers et al., 2000; Sun, 2002; Wen et al., 2014). For nearly 20 years, it has become a hot spot in the study of adaptive evolution (Favre et al., 2015; Yang et al., 2019; Zhang et al., 2019). Since 50 Ma (million years ago), this area has undergone long-term and huge geological changes, forming the highest and largest plateau in the world, with an average elevation of more than 4,000 meters (Royden et al., 2008; Deng and Ding, 2015). Especially, since the Quaternary, the QTP and its surrounding areas have been directly affected by drastic geology and climate changes (An et al., 2011; Liu and Dong, 2013; Li et al., 2015; Ren et al., 2018). Although its habitat is extreme and special, many unique taxa (*Rhodiola, Rhododendron, Berberis, Pedicularis, Hippophae*, etc) have evolved in the special environment. Organisms growing in the QTP undergo multiple evolutionary pressures than low-altitude species, including low temperature and oxygen, poor soils, and strong ultraviolet (UV) radiation (Sun, 1996). How plants adapt to extreme habitats at high altitudes has always been a fascinating and challenging problem.

With the development of high-throughput sequencing technology, some studies have tried to uncover the adaptation mechanism of plants to high-altitude habitats on the basis of the genome. Previous studies revealed some candidate genes related to high-altitude adaptation, as well as specific whole-genome duplication events (Zhang et al., 2016; Yang et al., 2020), LTR retrotransposons proliferation (Zhang et al., 2019; Geng et al., 2021; Feng et al., 2022), expanding gene families associated with DNA damage repair (Chen et al., 2019; Wang et al., 2021) and mutation of the flowering protein locus (Geng et al., 2021), which may contribute to the high-altitude environment adaption.

However, complete genomes of woody plants are rarely sequenced and published, especially for those at altitudes above 4,000 meters. In addition, existing studies mainly focused on the discovery of the adaptive evolution of specific genes in response to extreme ecological factors (genes involved in altitude adaptation: hypoxia response, eg: *PKLR, SRF*; DNA damage repair, eg: *DRT102, TFB1*; UV-B tolerance, eg: *SCC3, XRCC4*; abiotic stress resistance, eg: *Hsp70, MYB*; reproduction pathways and cold tolerance, eg: *FmHd3a, FmFT*) (Zhang et al., 2016; Geng et al., 2021; Mao et al., 2021). Although the role of WGD and the proliferation of TE have also been concerned, how they drive organisms to adapt to higher habitats remains unclear. Especially, whether woody plants at higher altitudes have similar or other adaptive mechanisms needs more works.

**Figure 1:**
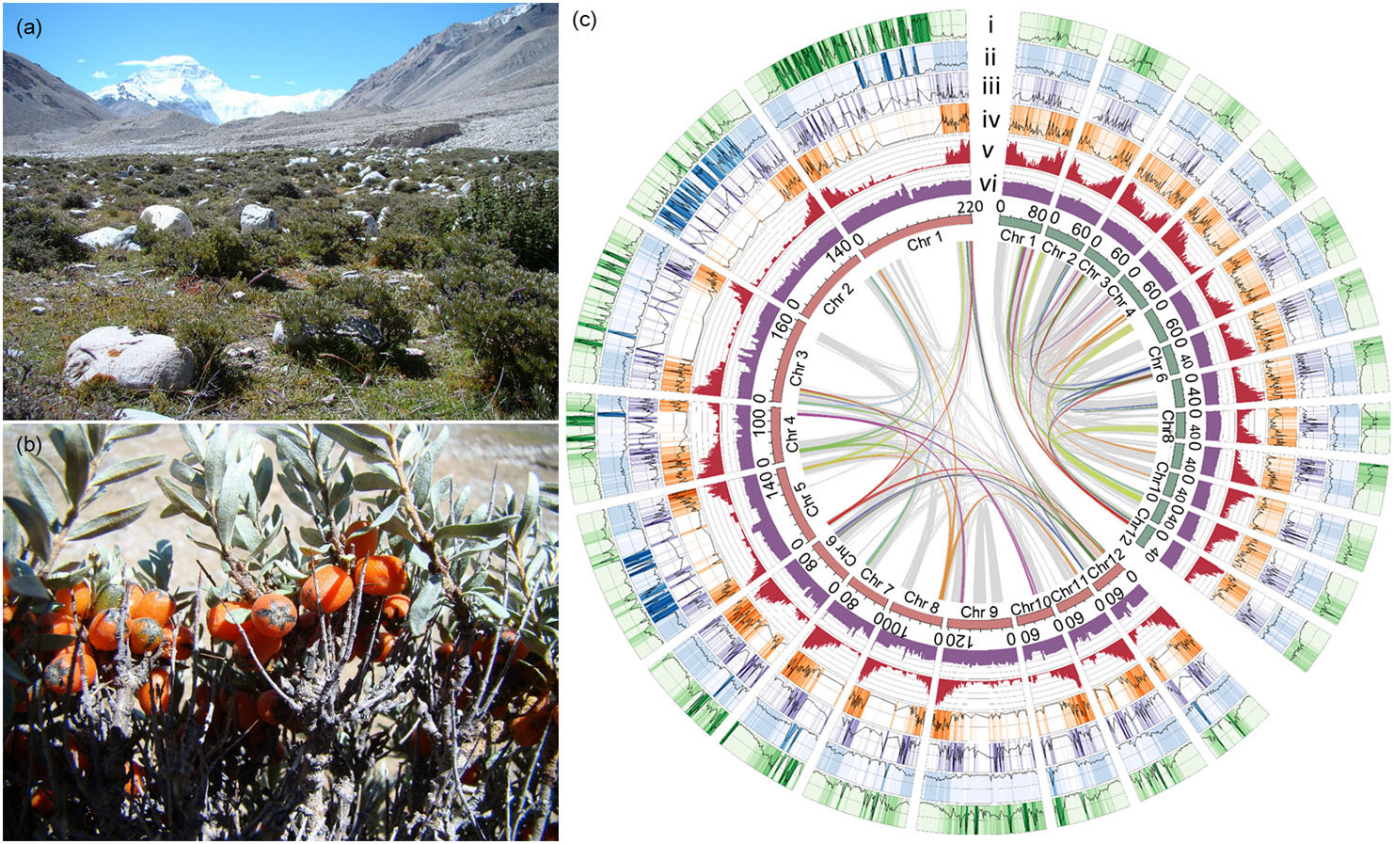
Habitat, morphological characteristics, and genomic features of ”*H. tibetana.* **(A)** High altitude habitat of ”*H. tibetana*; **(B)** Mature ”*H. tibetana* fruits on the tree; **(C)** Circos diagram of the chromosome-level ”*H. tibetana* (red inner circle on the left) and ”*H. rhamnoides* (green inner circle on the right) genome feature. Chr: chromosome. Window size: 1 Mb with non-overlapping. From outside to inside: i, percent of total ”*Gypsy* (0-100%); ii, percent of total ”*Copia* (0-100%); iii, intact ”*Gypsy* number (0-8); iv, intact ”*Copia* number (0-20); v, gene count (0-100); vi, GC content (0-100%). The gray line in the circle is the collinearity area between species, and the color line represents the fragments of 4 copies within species.

The genus *Hippophae* (Elaeagnaceae), commonly known as sea-buckthorn, consists of dioecious shrubby or woody species which are typical alpine plants mainly distributed in the Qinghai-Tibet Plateau (Lian et al., 1998). Due to its high drought-tolerance and wind-sand-tolerance, *Hippophae* species are widely used for soil/water conservation and phytoremediation of mining wasteland. As an important resource plant, large amounts of *Hippophae* plants are planted in northwestern China for desert greening and food ingredients (Lu et al., 2008). As one species of *Hippophae*, *H. tibetana* is a pioneer plant that can live in extreme habitats owing to a remarkable adaptability to cold, drought, extremely low supplies of nitrogen, and powerful UV-B ultraviolet light stress (280-315 nm; Körner, 2003), and is also one of woody plants grown at highest altitude in the world (**Figures 1A, B**), reaching to ~3,000-5,200 meters above sea level (a.s.l.). The morphological characteristics and high altitude habitat of *H. tibetana* are illustrated in **Figures 1A** **, B**. Nowadays, the genome of *Hippophae rhamnoides*, a relative of *H. tibetana*, has been published (Wu et al., 2022; Yu et al., 2022), *H. rhamnoides* is a regular or small tree distributed at lower altitude ranged from 490-3,700 meters a.s.l. (Huang and Yu, 2006). It is possible to reveal how plants adapt to more extreme habitats at higher altitude by comparing the genomes of the two species. In this study, we sequenced and assembled a high-quality genome of *H. tibetana*, and performed a detailed analysis of the genome evolution. The results could provide insights into evolution of the alpine plants.

## Materials and methods

### Sample collection

One wild *H. tibetana* individual used for *de novo* assembly was collected from Ganzi Tibetan Autonomous Prefecture, Sichuan Province, China (N 100.1056, E 29.1806). Total genomic DNA was isolated from the fresh leaves using the modified cetyltrimethylammonium bromide (CTAB) method (Doyle and Doyle, 1987), then dissolved in 50 *μ*L of sterilized water and kept at –30°C. Young leaves were collected from another individual seedlings from the same population for Hi-C sequencing. For RNA sequencing, seeds of the same plant used for genome assembly were selected and cultured. After 4 weeks of growth, young leaves, stems and roots were collected. All samples were rinsed with Milli-Q water and immediately stored in liquid nitrogen.

### Library construction and sequencing

A 250 bp short insert libraries was constructed and sequenced in 150 bp paired-end mode on the MGISEQ-2000 platform. The adapters and low-quality reads (reads with N bases more than 1% and the number of bases with Q-score ≤20 more than 10%) in raw data were removed using SOAPnuke 1.5.6 software (Chen et al., 2018) with the parameters set as follows: -n 0.01, -l 20, -q 0.1, -i, -Q 2, -G, -M 2, -A 0.5, -d. After filtration, 98.05 Gb high-quality data were retained, representing 68.47× genome coverage. Three 20 kb insert circular consensus sequencing (CCS) libraries were constructed and sequenced using a PacBio Sequal II platform for HiFi (high-fidelity) sequencing. The Hi-C library was constructed according to a published protocol (Durand et al., 2016). In brief, 2 g of young leaves was cross-linked *in situ* in 1% formaldehyde solution. Chromatin was extracted and digested with MboI (New England Biolabs), and the DNA ends were labeled, biotinylated, diluted, and randomly ligated. The DNA fragments were enriched and quality-checked to ensure that they were suitable for library preparation. Three sequencing libraries were constructed and sequenced on the MGISEQ-2000 platform in 150 bp paired-end mode. Transcriptome sequences were generated from a pool of mixed tissues including roots, stems and spear leaves. Raw polymerase reads were produced by PacBio Sequel platform, and then processed by IsoSeq technology through SMRT method (https://www.pacb.com/smrt-science/smrt-sequencing/) to obtain full-length non-chimeric reads.

### Genome assembly

Before *de novo* assembly, Jellyfish (Marçais and Kingsford, 2011) and Genomescope v.1.0 (Vurture et al., 2017) were employed to calculate the frequency of K-mer (k = 17) based on short reads data, and the genome size was estimated using a method based on K-mer distribution (Marçais and Kingsford, 2011). Contigs from CCS clean reads were assembled by Hifiasm (v0.15.1) (Cheng et al., 2021) with default parameters. Furthermore, PurgeDups (v.1.2.3) (Guan et  al., 2020; https://github.com/dfguan/purge_dups) was used to filter redundancy and generate the final haploid assembly. Scaffolding was performed using Hi-C based information, in which Hi-C reads were aligned to the assembled draft genome by Juicer v1.6 (Durand et al., 2016) and contigs were mapped onto chromosome-level scaffolds by 3D-DNA (Dudchenko et al., 2017) under default parameters. Manual checking and refinement of the draft assembly were carried out *via* Juicebox Assembly Tools (https://github.com/aidenlab/Juicebox, v1.1108). Aligned with the embryophyta_odb10 database (n = 1614), the completeness of the final assembly was evaluated using BUSCO v5.1.2 (Simao et al., 2015) with default parameters.

### Annotation of repetitive elements

Repetitive elements within the *H. tibetana* genome were identified using a combination of homology-based and *de novo*-based approaches. For homology-based annotation, RepeatMasker v4.0.7 (-nolow -no_is -norna -engine ncbi -parallel 1) (Chen, 2004) and RepeatProteinMask v4.0.7 (-engine ncbi -noLowSimple -pvalue 0.0001) (Bergman and Quesneville, 2007) were used to align the genome sequences against the Repbase database v21.12 (Jurka et al., 2005) to identify and classify different repetitive elements. For *de novo*-based prediction, RepeatModeler v1.0.8 (http://repeatmasker.org/RepeatModeler/) and LTR Finder (v1.0.6) (Xu & Wang, 2007) with default parameters was firstly used to construct the library and Repeatmasker was used for predicting repetitive elements. In addition, tandem repeats were predicted using Tandem Repeats Finder v4.09 (Price et al., 2005). The intact LTR-RTs in the *H. tibetana* (*μ*=7.06 × 10^−9^ per site per generation), *Hippophae rhamnoides* (*μ*=7.15 × 10^−9^ per site per generation), and *Ziziphus jujuba* (*μ*=6.20 × 10^−9^ per site per generation) genome were identified by TEsorter and the parameters are as follows: -db rexdb-plant -p 20 -cov 20 -eval 0.001. We used protein for the alignment and phylogenetic analysis and the timing of their insertion was estimated using LTR_retriever (Ou and Jiang, 2018). Phylogenetic analyses were carried out focusing on proteins of the reverse transcriptase domains of both Ty1-*Copia* and Ty3-*Gypsy* LTR-RTs for both the *Copia* and *Gypsy* family, the three species reverse transcriptase domains were merged and ~1000 paralogs were randomly extracted. The ~1000 multiple sequences alignments were carried out using MUSCLE v3.7 (Edgar, 2004). Phylogeny construction based on the maximum-likelihood (ML) method using IQ-TREE (v1.6.12) (Nguyen et al., 2015), with the best-fit evolutionary substitution model was evaluated using ModelFinder (Kalyaanamoorthy et al., 2017).

### Gene annotation

Gene annotations were performed by integrating evidence from homology-, *De novo*- and transcriptome-based information. In the homology-based approach, homologous proteins from *Arabidopsis thaliana*, *Oryza sativa*, *Cannabis sativa*, *Fragaria viridis*, *Z. jujuba*, *Pyrus pyrifolia*, were aligned against the *H. tibetana* genome using TBLASTN (Camacho et al., 2009), and the gene structure was predicted from these alignments by Exonerate v2.2.0 (Slater and Birney, 2005). *De novo* gene prediction was performed using a combination of AUGUSTUS (Stanke et al., 2006) and SNAP (Johnson et al., 2008) with default settings. The transcriptome data was aligned to the genome for predicting gene structure by BLAT. The tool MAKER2 (Holt and Yandell, 2011) was used to integrate the evidence for gene models.

Gene function annotation was done based on sequence similarity and domains conservation. First, the protein coding genes were annotated by homologous searches against public databases using BLAST v2.2.31 (E-value< 1e^-5^), including SwissProt (Boeckmann et al., 2003), KEGG (Kanehisa et al., 2012), GO (Ashburner et al., 2000), TrEMBL (Bairoch and Apweiler, 2000), InterPro (Zdobnov and Apweiler, 2001) and NR (https://www.ncbi.nlm.nih.gov/protein/). Subsequently, the best match from the alignment was used to represent the gene function by using some custom scripts. Second, we combined with application of InterProScan (51.0-55.0) (Jones et al., 2014) searching against the following databases to identify the motif and domain: Pfam (Bateman et al., 2000), PANTHER (Mi et al., 2017), SUPERFAMILY (Wilson et al., 2009), SMART (Schultz et al., 2000), PRINTS (Attwood et al., 2000) and ProDom (Corpet et al., 1999). The BUSCO was used to assess the completeness of the coding gene prediction for *H. tibetana* with default parameters. We mapped the gene density, GC content, fragmentary/intact *Gypsy* density, fragmentary/intact *Copia* density and chromosome synteny onto 12 chromosomes using the CIRCOS tool (http://www.circos.ca) (Krzywinski et al., 2009).

### Gene family evolution analysis

The protein-coding genes of H. tibetana and 12 additional plants species were used to identify orthologous gene groups, including Hippophae rhamnoides, Amborella trichopoda, A. thaliana, C. sativa, F. viridis, M. notabilis, O. sativa, Populus trichocarpa, Prunus persica, Rhamnella rubrinervis, Vitis vinifera, and Z. jujuba were downloaded. To perform the gene family analysis, orthogroups of the 13 species were identified using OrthoFinder (v2.3.11) with default parameters (Emms and Kelly, 2019). The single-copy genes were used for further phylogenetic analysis. For a certain gene-family in one species (paralog), it specially exists in this species. Other species have no gene clustered in the gene-family. The single-copy orthologs among the 13 species were aligned using MUSCLE v3.7 (Edgar, 2004) with default parameters and then the aligned protein sequences were reversely translated into codon sequences. The alignments were then joined into a super alignment matrix for phylogeny construction based on the maximum-likelihood (ML) method using IQ-TREE (v1.6.12) (Nguyen et al., 2015), with the best-fit evolutionary substitution model was evaluated using ModelFinder (Kalyaanamoorthy et al., 2017). Divergence time for each node in the phylogenetic tree was estimated with using MCMCtree implemented in PAML package v4.8a (Yang, 2007) with the following parameters: -nsample 100000, -burnin 500000. The time correction points were obtained from TimeTree database (http://www.timetree.org) (Kumar et al., 2017): 56 ~ 93 million years ago (Ma) for R. rubrinervis and Z. jujuba, 107 ~ 135 million years ago for Z. jujuba and V. vinifera, 1.73 ~ 1.99 million years ago for V. vinifera and A. trichopoda.

The time-calibrated phylogenetic tree was used for accessing gene family expansions and contractions by the CAFÉ 4.2.1 program (Han et al., 2013), using a random birth-and-death model with lambda potio. The corresponding *P*-value in each lineage was calculated based on the conditional likelihood method and filtered threshold of *P*-value is 0.05.

### Genome collinearity analysis and WGD event identification

MCscan (Python version) (Tang et al., 2008) was used for the genomic analysis between *H. tibetana, H. rhamnoides, V. vinifera, Z. jujuba, and A. trichopoda*. The collinearity figure was drawn based on the gene collinear pair information between species by JCVI (https://github.com/tanghaibao/jcvi) or by Circos (Krzywinski et al., 2009). We first characterized the synonymous nucleotide substitutions on synonymous substitution sites (*Ks*) between the collinear genes inferred above. The values of *Ks* was estimated for each collinearity orthologous using the *WGDI* (Sun et al., 2021) program with Nei-Gojobori method implemented in the YN00 program in the PAML (4.9h).

Divergent evolutionary rates among plants affect dating ancestral events. Here, based on an approach that previous report developed (Wang et al., 2015; Wang et al., 2018), we performed evolutionary rate correction by aligning the peaks in different genomes corresponding to the core-eudicot common hexaploidization (ECH) event which was ~115–130 Ma (Jiao et al., 2012; Vekemans et al., 2012) to the same locations.

For evolutionary rate correction, under the assumption that the *H. tibetana* peak appears at k_H_ and the *V. vinifera* peak appears at k_V_, we can use the equation


$$r\;=\;\left(k_{H}-\;k_{V}\right)/k_{V}$$

to describe the relative evolutionary rate of *H. tibetana*. Then, rate correction was performed to discover the corrected rate k_H correction_ of *V. vinifera* relative to k_V_:

For the Ks between duplicates in *H. tibetana*, we defined the correction coefficient W_H_ as


$$k_{H}correction/k_{H}=\;k_{V}/k_{H}=\;W_{H}$$

thus, we obtained


$$k_{H}correction=\;k_{V}/k_{H}\times \;k_{H}=\;1/\left(1\;+\;r\right)\;\times \;k_{H}and\;W_{H}=\;1/\left(1\;+\;r\right)$$

(2) For the *Ks* between homologous genes from *H. tibetana* and *V. vinifera*, if the peak was located at K_H-V_, supposing the correction coefficient W_H_ in *H. tibetana*, we then calculated a corrected evolutionary rate


$$k_{H-V-correction}=\;W_{H}\times \;k_{H-V}$$

The different substitution rates of different species were calculated by the *Ks* analyse above. The equation is:


r=K/2T

where r is the substitution rate; K is the original observation value in *Ks* distribution reflecting the core-eudicot common hexaploidization; and T is the occurrence time of the core-eudicot common hexaploidization even.

### Adaptive selection in the genome

The values of *Ka* and *Ks* and the *Ka*/*Ks* ratio were estimated for each collinearity orthologous genes using the *WGDI* (Sun et al., 2021) program with Nei-Gojobori method implemented in the YN00 program in the PAML (4.9h). We got the *Ka*/*Ks* result for the two groups (group 1: *H. tibetana* vs *R. rubrinervis* and *Z. jujuba* vs *R. rubrinervis*; group 2: *H. tibetana* vs *Z. jujuba* a*nd H. rhamnoides vs Z. jujuba*). The GO functions of *H. tibetana, H. rhamnoides* and *Z. jujuba* protein-coding genes were annotated by homologous searches against GO (Ashburner et al., 2000) public databases using BLAST v2.2.31 (E-value< 1e^-5^). The binomial test (Watanabe and Hattori, 2006; Qiu et al., 2012) was used to identify GO categories with more than 20 orthologs that had an excess of nonsynonymous changes in either *H. tibetana* or *Z. jujuba* (or *Hippophae rhamnoides*) lineages.

Positive selection is biologically important in adaptations to the environment. To identify positive selections in the evolution of *H. tibetana*, orthologous genes from *H. tibetana*, *Hippophae rhamnoides, R. rubrinervis, Z. jujuba* and *C. sativa* were identified using OrthoFinder (v2.3.11). The single-copy genes (3,218 groups) were used for aligning by using MUSCLE v3.7 (Edgar, 2004) respectively. The genes under positive selection were estimated using the branch-site model of Codeml program (Codon Freq = 2) implemented in the PAML package v4.8a, with *H. tibetana* as the foreground branch and the other four species as background branches. Likelihood ratio test was used to identify positively selected genes (PSGs) (Likelihood ratio test, *P ≤* 0.05).

### SVs identification

We detected the SVs of *H. tibetana* and *H. rhamnoides* by using SyRI (Goel et al., 2019). Firstly, the genomes (in multi-fasta format) are aligned using the NUCmer utility v 4.0.0 (-c 500 -l 40 -g 200 -t 10). Secondly, SyRI takes genome alignments coordinates as input to identify SVs (-k –nc 20 -c cords.file -d delta.file -q H. tibetana.fa -r H. rhamnoides.fa -s show-snps).

### *Nodule inception* gene family evolutionary analysis

*NODULE INCEPTION (NIN*) gene family members were identified by BLAST searching homologous sequences in *H. tibetana*, *Z. jujuba* and *H*. *rhamnoides* genomes by setting the *Medicago* NIN (MTR_5g099060) protein as the query sequence (E-value: 1e^−5^). Full-length NIN were used to build a ML-based phylogenetic tree using RAxML 7.0.4 software (Stamatakis, 2006) to construct the ML phylogenetic tree (ML-Tree).

To inspect the protein domain differences between NINs and NLPs, NIN/NLP proteins from all species were subjected to perform protein motif analysis with meme suit (Bailey et al., 2009) using the following parameters: *-nmotifs 20*, *-mod* zoops, *-minw 6* and *-maxw 50*, and the results (.xml file) were visualized using TBtools (Chen et al., 2020). For this analysis, only seed plants were used to avoid the extreme sequence variation caused by a long divergence time.

### Aligning sex-specific regions to the homogametic sex data sets

A total of 80 *H. thibetana* samples were sampled from the four sites (Mt. Everest, Tibet; Datong, Qinghai; Maizhokunggar, Tibet; Xiahe, Gansu), including 10 male and 10 female plants in each site for RAD seq. The geographic distance between the four sampling sites was between 273 and 1683 km. During the flowering season (June in 2013), female and male individuals were identified by gynoecium and staminate flowers.

A mean of 1.89 Gb raw data was generated for each individual, the sequencing quality of Q20 and Q30 was 95% and 87% respectively. GC content was a little vibrate because of a small amount of chloroplast contamination. After quality control, a total of 110,619 Mb clean sequence reads were acquired from 150 bp paired-end sequencing (Insertsize: 500 bp) on the Illumina Hiseq 2000 platform (Illumina, San Diego, CA, USA), with an average of 1,382 Mb for each individual (minimum = 567,569; maximum = 15,706,831). The SNPs with MAF ≥ 0.05 and missing rate ≤ 0.2 and without imputation were used for GWAS. The Mixed linear model (MLM) analysis was conducted for GWAS using GAPIT software (Lipka et al., 2012). To avoid the false positive, the population structure (Q) and a polygene (K) were applied in GWAS. The total number of bi-allelic single nucleotide polymorphism was 2,486,852, and 2,072,124 SNPs were left after filtering. *H. tibetana* is known to have an XX/XY sex-determining mechanism. All putative sex-specific markers that passed this confirmation step were assembled into Contigs (RAD loci) with paired-end reads using Sequencher software (http://www.genecodes.com/). We further validated putative sex-linked RAD loci. First, we searched the female *H. tibetana* genome for putative male-specific RAD loci using BLAT on the UCSC Genome Browser (Kent, 2002; Meyer et al., 2013) and excluded any significant matches. Then, BLAST was used to search the NCBI nucleotide database for matches to exclude contamination.

## Results

### Genome assembly and quality assessment

A *H. tibetana* (2n = 2x = 24) wild individual in Litang County, Sichuan (China) was used for whole-genome sequencing. Totals of 152 Gb of clean Illumina short-read data (~100× coverage), 98.05 Gb of PacBio HiFi reads data (68.47× coverage), and 228 Gb of BGI-sequenced Hi-C data (~159× coverage) were generated. A total of 531.5 million unique mapped Hi-C PE reads was detected and the Hi-C effective data was accounted for 25.81%. After obtained the assembly from Hifiasm (v0.15.1) and PurgeDups (v.1.2.3), contigs were clustered, sorted, orientated, and finally assembled onto 12 chromosomes with genome size of 1,452.75 Mb (96.04% of the final assembled 1,512.72 Mb genome) and heat maps are drawn accordingly (**Figure S2**). This genome assembly size was comparable to the size estimated by a K-mer-based method (1,432.13 Mb) (**Figure S1**). The assembly genome size is consisting with the DNA content of its related species (2.32-3.88 pg/C) that was previously determined with tissue using flow cytometry (Zhou et al., 2012) and is much larger than other Elaeagnaceae species (Mao et al., 2021; Wu et al., 2022; Yu et al., 2022). The Contig N50 and N90 of assembly genome reaches 74.31 Mb and 24.93 Mb and the scaffold N50 and N90 reaches 123.42 Mb and 71 Mb respectively (**Table 1**). Genome quality assessment was evaluated by Benchmarking Universal Single-Copy Orthologs (BUSCO) (Simao et al., 2015), and revealed that the genome completeness reached 95.5%, a total of 1,542 expected embryophyta genes were identified in *H. tibetana* genome (**Table S1**), suggesting that the genome assembly is of high quality.

**Table 1:** Statistics of the genome assembly and annotation.

Genome Features	*H. tibetana*	*H. rhamnoides* ssp*. sinensis*	*H. rhamnoides* ssp*. mongolia*
Assembly strategy	PacBio CCS +Hi-C	PacBio CLR+ Hi-C	PacBio CLR+ Hi-C
Estimated size (Mb)	1,432	749	978
Assembly size (Mb)	1,452.75	730.46	849.04
Number of Contigs	1,409	1,386	3,642
Maximum Contig length (Mb)	123.41	82.44	92.33
Contig N50 (Mb)	74.31	2.81	2.15
Contig N90 (Mb)	26.14	0.373	–
GC content (%)	29.37%	30.07%	30.07%
Repeat content (%)	72.18%	47.48%	67.81%
Number of protein-coding genes	33,367	30,812	30,864
Complete BUSCOs	95.54%	97.60%	89.65%

Combining *De novo*-, transcriptome- and homology-based predictions, 33,367 protein-coding genes were predicted from the genome overall. The number of gene models is comparable to those of other Elaeagnaceae species (Mao et al., 2021; Wu et al., 2022; Yu et al., 2022). Among all the predicted genes, 32,059 genes could be annotated to the functional database, see **Table S2**. The intersection of functional annotations among the five databases is shown in **Figure S3**. There are 21,081 genes with functional annotations in these five databases. The BUSCO score indicates that 95.0% of complete core orthologs were identified, suggesting that the gene annotation is of high quality. Otherwise, 15,143 non-coding RNA genes were identified in the genome, including 6,823 rRNAs, 5,221 tRNAs, 164 miRNAs, and 2,935 snRNAs (**Table S3**). An overview of the distribution of the predicted gene models in the genome is shown in **Figure 1C**.

### Genome annotation and recent burst of LTR-RT

After removing the overlapping regions between various methods, the remaining nonredundant repeated sequences accounted for 72.18% of the genome size (**Table S4**), and transposable elements (TEs) were the dominant components (69.52%, **Table S5**). The TEs including DNA transposon elements (DNA, 2.77%), long interspersed nuclear elements (LINEs, 0.54%), short interspersed nuclear elements (SINEs, 0.03%), long terminal repeats (LTRs, 64.85%), and elements of unknown classifications (2.05%) (**Table S5**).

**Figure 2:**
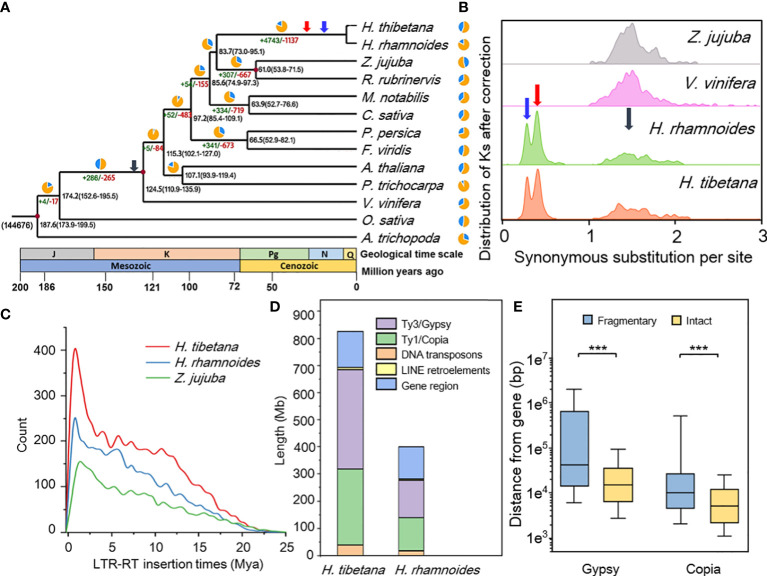
**(A)** Phylogenetic tree constructed of ”*H. tibetana* and 12 other representative species. Divergence time estimation and gene family changes among 13 plant species. *A. trichopoda* was used as a basal species in the phylogenetic tree. The black number at each node denotes estimated divergence time from present (million years ago). The number at the root (144,676) denotes the total number of gene families, and the green/red numbers around each branch denote gene family gain/loss number. The red dot as the corrected time of the Timetree website; **(B)** Synonymous nucleotide substitutions (*Ks*) distribution in syntenic blocks after correction. Syntenic blocks (involving ≥10 colinear genes) within a species or between two species were collected, and the median Ks values for each block were obtained. Distribution of *Ks* between paralogs or orthologs of ”*H. tibetana - ”H. tibetana* showed HRT (blue arrow) and HAT (red arrow) represent the two tetraploidization events, ECH (grey arrow) represents the core-eudicot common hexaploidization (WGT, γ event); **(C)** Distribution of LTR retrotransposon insertion time of ”*H. tibetana, H. rhamnoides* and *Z. jujuba*; **(D)** The proportions of TEs in ”*H. tibetana* genome; **(E)** Distance between intact/fragmentary LTR (*Gypsy*/*Copia*) and gene, *** significant at < 0.001 level.

TE contents are highly variable within eukaryotes and generally comprise the important part of plant genome, and the summation of TE show a positive correlation with plant genome size (Wendel et al., 2016). LTR-RTs possessed the most proportion (64.85%) of repetitive elements in *H. tibetana*, and a total of 6,911 intact LTR-RTs in *H. thibetana*, 4,601 in *H. rhamnoides* and 3,004 in *Z. jujuba* genome were identified. Meanwhile, by using the substitution rate values calculated by the Ks analyse, both insertion time of *H. rhamnoides* (~1 Ma) and *H. tibetana* (~1 Ma) were more recent compared with *Z. jujuba* (~1.5 Ma) (**Figure 2C**).

The Ty3/*Gypsy* and Ty1/*Copia* elements were the two main types of LTR in *H. tibetana*, accounting for 25.39% and 19.33%, respectively (**Figure 2D**; **Table S5**). Phylogenetic analysis of intact Ty3/*Gypsy* and Ty1/*Copia* supergroups showed that the branches of *H. tibetana* were short and clustered distribution, suggesting that recent burst of Ty1/*Copia* and Ty3/*Gypsy*, indicating that there was a recent TE amplification of *H. tibetana* (**Table S6**; **Figures 2D**, **S4**). Intact *Gypsy*/*Copia* is closer to genes than fragmentary *Gypsy*/*Copia* (**Figure 2E**; chi-square test, P<0.05). Surprisingly, we identified a DNA transposon gene family with as many as 141 members in the *H. tibetana* genome (**Figure S5**). *H. tibetana* is almost twice the genome size of *H. rhamnoides* (**Table 1**), but the number of gene models and the total length of exons and introns are comparable to *H. thibetana* (**Figure S6**). The primary cause is that the length of intergenic regions in *H. thibetana* genome was significantly greater than that in *H. rhamnoides* genome and the majority of TEs were located in those (**Figure 2D**). *H. tibetana* is consist with this pattern, and possessed both large genome size and larger proportion of TEs. The recent proliferation of LTR-RTs in the genome of *H. thibetana* may promote its genome evolution and play an important role in adapting to high-altitude habitats alone after its differentiation with *H. rhamnoides*.

### Gene family evolution and phylogenetic analysis

After analysis of the gene family, 241,829 genes from the 13 species were grouped into 22,503 gene families (**Figure S7**). Venn diagram depicting the number of shared and specific gene families among *H. tibetana* and three representative plants (*Z. jujuba, V. vinifera* and *H. rhamnoides*). We identify 10,044 homologous gene families shared by these 4 species, and 1,319 gene families were specific to *H. tibetana* and *H. rhamnoides*, and 713 were found only in *H. tibetana* (**Figure S8**). *H. tibetana* shows more genes in common with *Z. jujuba*, *R. rubrinervis*, and *C. sativa* than with other species (**Table S7**). Among the orthologous genes, 581 genes were identified as single-copy genes in these species, which were used for inferring the evolutionary relationships. As shown in the phylogenetic **(** **Figure 2A** **)**, *A. thaliana, P. trichocarpa*, *V. vinifera*, *O. sativa*, and *A. trichopoda* diverged from one another earlier than *M. notabilis, C. sativa, P. persica*, and *F. viridis*, diverged from each other, and *Z. jujuba*, together with *R. rubrinervis* cluster as a sister is most closely related to *H. tibetana*. According to the fossil record information, *H. tibetana* separated from the Rhamnaceae approximately 83.7 Ma (**Figure 2A**). The diverged time of Elaeagnaceae and other Rosales species at the beginning of the Cenozoic era. According to previous studies and reports, the rapid diversification of species in this family only occurred in the Miocene which was less than 23 Ma (Jia and Bartish, 2018).

### Recent whole-genome duplication and diploidization

To explore whole-genome duplication, we compared the *H. tibetana* genome with that of three representative or related plant species: *Z. jujuba* (2n=24)*, V. vinifera* (2n=38) and *H. rhamnoides* (2n=24) (**Table S5**). Synonymous substitution sites rate (*Ks*) between the collinear genes were calculated paralogs in *Z. jujuba, V. vinifera* and *H. tibetana.* There were three collinear blocks peaks of *H. tibetana* were detected, and located at 0.329 ± 0.001, 0.502 ± 0.002 and 2.019 ± 0.021 after data fitting (**Figure 2B**). Combining of our present analyses and the research of *H. rhamnoides* genome (Yu et al., 2022), indicated that there were two lineage-specific polyploidization events had occurred in the genus *Hippophae*, and the two rounds of polyploidization occurred within a relatively narrow timeframe (**Figure S9** **)**. In addition to the core-eudicot common hexaploidization (whole genome triplication, WGT; γ event, grey arrow, **Figure 2A**), two prominent peaks were identified (*Hippophae* recent tetraploidization, HRT: 18.7-21.2 Ma, blue arrow; *Hippophae* ancient tetraploidization, HAT: 28.6-32.4 Ma, red arrow, **Figure 2A**) in the *K*s profiles of the *H. tibetana* genome, but unlike most other apricot, both HRT and HAT occurred at a *K*s value less than 1. We calculated the substitution rates to 7.06E^-09^, 7.15E^-09^, 6.20E^-09^ and 5.16E^-09^ for *H. tibetana, H. rhamnoides*, *Z. jujuba*, and *V. vinifera* respectively.

Even after two rounds of chromosomal breakage and fusion events, the 1:1 syntenic blocks between *H. rhamnoides* (**Figure S10** **)** and *H. tibetana* and the number of *Hippophae* chromosome number remained to be 12. The *H. tibetana* specific WGD event was also supported by the 1:4 syntenic blocks between *Z. jujuba* and *H. tibetana* (**Figure S11** and **Figure 1C**). This is also consistent with previous observations that there was no WGD event occurred in jujube (Liu et al., 2014).

Comparative analyses revealed conserved synthetic with part of chromosomal rearrangements between the genomes of *H. rhamnoides* and *H. tibetana*, a total of identified 1,713 structural variants (SVs) was identified (**Figure S12** **)**, and large chromosomal inversion were detected on Chr1, Chr2, Chr6, Chr11, and Chr12.

### Genome evolution in adaptation and survival

**Figure 3:**
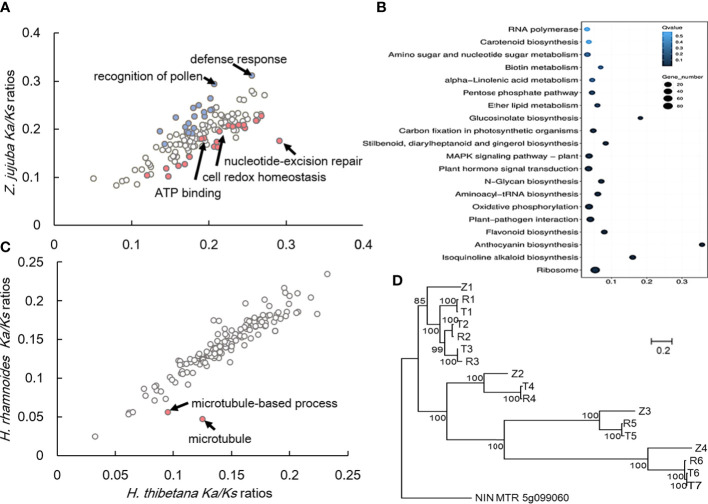
Rapid evolution gene and specific gene sets (expansion-related genes and genes under positive selection) in ”*H. tibetana.* **(A)** GO categories with putatively accelerated (”*P*< 0.05, binomial test) nonsynonymous divergence in ”*H. thibetana* (red) or *Z. jujuba* (blue) are highlighted. **(B)** Functional enrichment of the expansion genes, the gene number and the signification of each GO term was indicated by size and color of dot; **(C)** GO categories with putatively accelerated (*P*< 0.05, binomial test) nonsynonymous divergence in ”*H. thibetana* (red) or *H. rhamnoides* are highlighted; **(D)** Maximum-likelihood phylogenetic tree of *NODULE INCEPTION* in *Z. jujuba, ”H. rhamnoides and ”H. tibetana*. The scale bar shows the expected number of amino acid substitutions per residue.

As sessile organisms live in high altitude area, *H. tibetana* have to cope with recurring stress such as frequent low nocturnal temperatures, intense solar ultraviolet, low carbon dioxide concentration. To clarify the evolutionary adaptation of *H. tibetana*, the time-calibrated phylogenetic tree was used for accessing gene family expansions and contractions by the CAFÉ 4.2.1 program (Han et al., 2013). There were 186 and 94 gene families (1,113 genes Gain and 870 genes loss) were significantly expanded and contracted, respectively (P < 0.05; **Figures 2A**, **3B**), which indicated that there were more *H. tibetana* gene families experienced expansion rather than contraction during adaptive evolution. After GO enrichment analyses, genes were enriched in “oxidoreductase activity (GO: 0016491),” “cellulose biosynthetic process (GO: 0030244),” and “protein phosphorylation (GO: 0006468)”.

After filtering out organelle genes, 3,101 genes of the *H. tibetana* specific gene families were identified, and Gene Ontology annotation terms mainly containing “response to ultraviolet (UV) radiation,” “Plant-pathogen interaction,” “MAPK signaling pathway – plant,” “Phenylpropanoid biosynthesis,” “Flavonoid biosynthesis,” “Phenylalanine metabolism,” and “Nitrogen metabolism”.

*Ka*/*Ks* ratios of nonsynonymous-to-synonymous substitutions for different GO categories revealed an enrichment of elevated pairwise *Ka*/*Ks* values in the high-altitude adaptation (Qiu et al., 2012). Analysis of *Ka*/*Ks* ratios in the lineages verified that genes with elevated *Ka*/*Ks* values in *H. tibetana* were significantly (*P*< 0.05, binomial test) enriched for high altitude adaptation functions (**Table S8**; **Figures 3A, C**). Most of genes were enrich in “nucleotide-excision repair,” “cell redox homeostasis,” and “ATP binding”. On the contrary, GO categories with putatively accelerated nonsynonymous divergence in *Z. jujuba* (blue) were enrichment in “recognition of pollen” and “defense response”. After that, we detected genes evolving under elevated *Ka*/*Ks* values in either *H. tibetana* or *H. rhamnoides*. Only “microtubule-based process (GO: 0007017, process)” and “microtubule (GO: 0005874, component)” were identified as rapidly evolving (with elevated *Ka*/*Ks* values in *H. tibetana*) genes.

Furthermore, the positively selected genes compared with close relatives in the plant genome are usually considered to be related to adaptability (Fitch, 1970). To test the hypothesis that these rapidly evolving genes in *H. tibetana* have been under positive selection, we used the branch-site likelihood ratio test to identify positively selected genes (PSGs) in the *H. tibetana* lineages. In *H. tibetana*, a total of 249 genes were identified as positive selection genes by comparative analysis (P < 0.05), after GO enrichment analyses (**Table S9**; **Figure S13**), most of genes were enrichment in “protein binding (GO: 0005515),” “cellular response to DNA damage stimulus (GO: 0006974),” “negative regulation of defense response (GO: 0031348),” “DNA repair (GO: 0006281),” “carbohydrate metabolic process (GO: 0030246)”. These processes are related to the adaptation to the extreme environmental conditions of high ultraviolet and low temperature in the high-altitude area of ​​the Qinghai-Tibet Plateau.

### Nodule inception gene family evolution of H. tibetana

Symbiotic nitrogen fixation provides a large amount of sustainable and environmentally friendly nitrogen for plants. Comparative analysis of NIN-orthologous regions within related species (*Z. jujuba*, *H. rhamnoides*, and *H. tibetana*). Three species exhibited different copy numbers within these regions; *Z. jujuba* exhibited 4, *H. rhamnoides* 6, and *H. tibetana* 7 (**Figure 3D**). Also, because *H. tibetana* is a pioneer species, it can survive on the bare ground where other plants cannot colonize. We deduced that this additional nitrogen-fixation-related gene was helpful for *H. tibetana* to colonize bare land first.

### Identification of sex-linked regions

**Figure 4:**
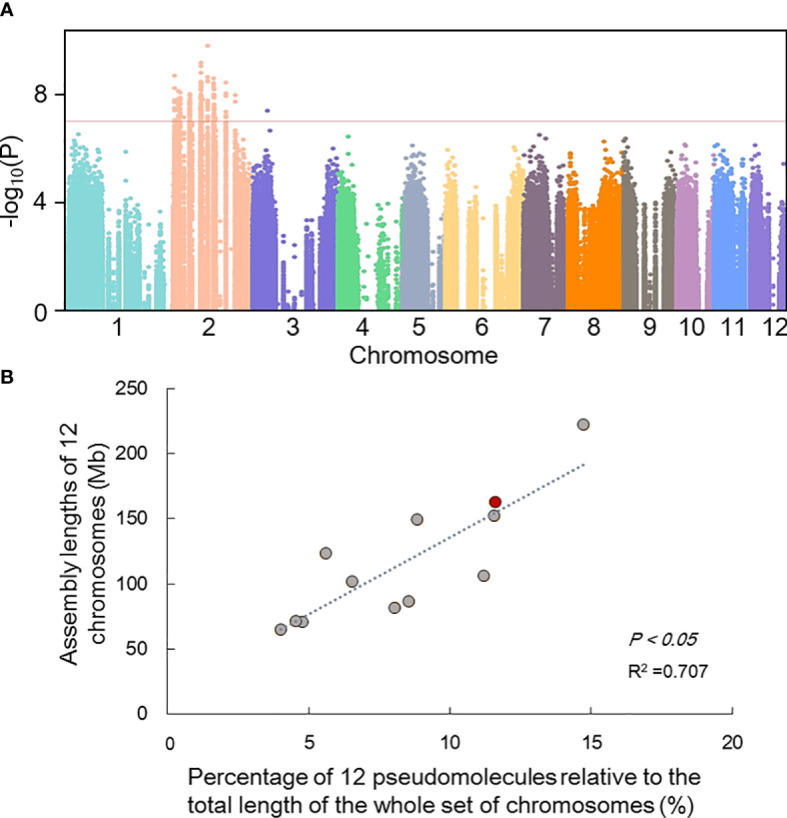
Preliminary inference of sex-linked regions. **(A)** Manhattan depicting significant SNPs identified using MLM model that showed association with gender. The red dotted line represents truncation criterion was set to - log10 *P*-value=7; **(B)** The correlation between the assembly lengths and observed physical lengths of all chromosomes. The red dot represents chromosome 2 of *H. tibetana*.

Screening of male-specific markers by comparative analysis of RAD-seq sequencing of 40 male and 40 female samples of known gender (distinguished according to morphology in reproductive season (**Figure 1B**; **Table S10**). RAD-seq paired-end reads was detected SNPs by *Stacks*1. 04 (Catchen et al., 2013). Genome-Wide Association Studies (GWAS) among 40 males and 40 females was performed and showed that the regions on Chr2 were related to sex **(** **Figure 4A** **),** which also supported by correlation between the assembly lengths and observed physical lengths of all chromosomes **(** **Figure 4B** **)** (Xing et al., 1989).

## Discussion

*H. tibetana* can live in extreme environments at 5,200 meters a.s.l. and is one of the highest distributed woody plants. Here, a chromosome-level genome of wild *H. tibetana* was assembled and annotated with contig N50 sizes of 74.31 Mb, which possessed high completeness, accuracy, and complexity. This is also the highest known woody plant genome (up to 5,200 meters above sea level). Although it has the same chromosome number with two related species (*Z. jujuba* and *H. rhamnoides*), the genome size of *H. tibetana* is 1,452.75 Mb and 41.56% larger than that of *H. rhamnoides* (Yu et al., 2022), and 69.87% larger than that of *Z. jujuba* (Huang et al., 2016). The comparison of *H. tibetana* and these related species revealed some new findings.

Studies of high-altitude plant genomes have revealed that some genes with special adaptive significances have been affected by positive selection and undergone rapid evolution. Especially, the genes involved with DNA damage repair, reproductive processes, and UV-B tolerance (Mao et al., 2021). We also found some interesting genes to undergo a rapid/slow evolution by comparing *H. tibetana* with *Z. jujuba*, which altitude is less than 1,700 meters (Chen and Chou, 1982). The expansion or positively selected genes involved in cellular response to DNA damage stimulus, Smc5-Smc6 complex, SUMO ligase complex and nitrogen compound metabolic process. These genes involved in adaptation to high altitude habitats have also been identified in previous studies (Zhang et al., 2016; Geng et al., 2021; Mao et al., 2021).

It is worth noting that compared with jujube, two rapidly evolving genes in jujube were found, which were enrichment in “recognition of pollen” and “defense response”. Since *H. tibetana* is a pioneer species, it can survive on bare land where few other plants survive. Compared with its relatives, it has fewer pollens from other competitors, so “recognition of pollen” genes rapidly evolving should be adaptive in jujube. In addition to the above genes, there are some genes that evolve rapidly relative to jujube were enrich in “nucleotide-excision repair,” “cell redox homeostasis,” and “ATP binding” (**Figure 3A**), suggesting that high-altitude habitats also have a significant impact on the basic life activities of organisms.

However, when comparing *H. tibetana* with *H. rhamnoides*, none of the genes were subject to significant positive or negative selection except microtubule-based process (GO: 0007017, process) and microtubule (GO: 0005874, component) (**Figure 3C**). Microtubule and microtubule-based process genes were considered to be related to plant growth and development (Hashimoto, 2003). We guess that they are likely to be the key genes that determine the evolution from tree to shrub to adapt to higher altitudes. We notice that the average altitude of *H. tibetana* is higher ~1,500 meters than that of *H. rhamnoides* (Huang and Yu, 2006). The fact that only two GO categories have undergone fast/slow evolution is interesting. These two species live in such different environments that it is hard to believe that only two GO categories are subject to significant positive selection. This could mean that the forces of natural selection may be acting more on other aspects of the genome than changing the amino acid sequence of proteins, such as gene number, gene regulatory regions, etc.

The significant fluctuation of gene family copy number is usually related to the adaptive evolution of species (Sudmant et al., 2010). We noticed that the genome of *H. tibetana* contains 2503-2555 more genes than that of *H. rhamnoides*. Some of these genes were from single gene duplication, and most genes were originated from WGD. A special gene doubled in the former way was found in *H. tibetana* (**Figure 3D**). It was related to nitrogen fixation as the previous study found in the *H. rhamnoides* (Soyano et al., 2013; Liu and Bisseling, 2020). We found that in the *H. tibetana*, the key nitrogen fixation gene increased by one copy through tandem duplication, and that at least at the seedling stage, the transcript level these two genes in *H. tibetana* was 2.43~2.53 times than that in *H. rhamnoides* (*H. tibetana*: T1-T7, *H.* rhamnoides: R1-R6; **Figure 3D**). As described above, the habitat of *H. tibetana* is very extreme and harsh, especially prefers bare land after glaciers retreat, such habitats are deficient in available nitrogen. We proposed that the genome evolved nitrogen fixation adaptively by increasing the copy number of genes.

Obviously, WGD is the source of gene increase (De Smet and Van de Peer, 2012), and is considered to be an important way of speciation and adaptation to disturbed habitats (Soltis and Soltis, 1999; Jiao et al., 2011). Plants with known genomes on the Qinghai Tibet Plateau, eg: *Lepidium meyenii* and *Megacarpaea delavayi*, have experienced independent WGD (Zhang et al., 2016; Yang et al., 2020). In this study, we found that the *H. tibetana* genome experienced two WGD events at 18.7-21.2 Ma (HRT) and 28.6-32.4 Ma (HAT), respectively. Interestingly, after HAT and HRT, the genome of *H. tibetana* went through the process of chromosome breakage and fusion, and then the chromosome returned to its ancestral number (2n=24). As can be seen from **Figure 2A, B**, these events occurred before the differentiation of *H. tibetana* and *H. rhamnoides*, that is, they experienced these important processes together, and then went to different evolutionary paths respectively. We noted that the former has 2,503-2,555 genes more than the latter, and the genome size is 41.56%-49.72% larger than *H. rhamnoides*. Through functional enrichment of genes generated by WGD, it is found that these extra genes participate in the basic process of life activities. We speculate that this may be related to the drastic changes in the environment in a short time at a very high altitude, for example, in the area of 5,000 meters a.s.l., the temperature difference in one day (24 hours) can reach 20°C (Zhang and Yang, 2013), which means that cells need greater biochemistry plasticity to respond. Studies have shown that more gene redundancy can provide greater plasticity (Mattenberger et al., 2017).

Compared with the *H. rhamnoides* genome, one the most prominent characteristics of the *H. tibetana* genome is to contain more TE elements (**Figure 2D**), which is the main reason for the difference in size between the two genomes. TE outbreaks have also been widely found in high-altitude plant genomes, and it is believed that rapid proliferation of repetitive elements in *C. lasiocarpa* may play an important role in promoting its genome evolution (Feng et al., 2022). However, the relationship between high-altitude extreme habitats and TE still remains unclear. As a mobile element, the activity of transposable elements increases the variation of genome and can generate mutations rapidly (Kidwell and Lisch, 1997; Wessler, 2006), which is of great significance for alpine organisms to respond to environmental changes (Wos et al., 2021). Compared with the lower altitude area, alpine habitats are more susceptible to climate and other factors. On the Qinghai-Tibet Plateau, the greater impact used to happen in habitats with higher altitude, especially since the Quaternary, it has been affected by the repeated fluctuations of the glacial and interglacial periods (Shi, 2002; An et al., 2011; Yan et al., 2021). More variation in genome often means higher genetic diversity and therefore evolutionary potential. Although since 0.5 Ma, the effective population size of *H. tibetana* has continued to decline (Figure S14), this species has still widely distributed, perhaps due to previously emerging genomic adaptations.

The transposon proliferation time of *H. tibetana*, *H. rhamnoides*, and jujube is around 1~1.5 Ma (**Figure 2C**), suggesting that this is a parallel response stimulated by the environment, when the QTP enters freezing from this period circumstances and times of environmental upheaval (Shi et al., 1998; Zhou et al., 2011; Zhao et al., 2020). We noticed that the transposon size in the *H. tibetana* genome is the largest, following by *H. rhamnoides*, and then the species at the lowest altitude, *Z. jujuba*, has the smallest transposon size (or is less removal and more fragments remaining). How the activity of the TEs described above is linked to more dramatic environmental variability needs more works, and the activity of TEs can be induced by environmental and population factors and in particular by stresses in various organisms has been confirmed by many studies (Capy et al., 2000; Wos et al., 2021). Also, we detected a burst of DNA transposable elements that are only present within the *H. tibetana* (**Figure S6**). These results suggest that the extreme habitat of *H. tibetana* is the cause of it has more TEs.

By analyzing the distribution of transposons in the *H. tibetana* genome, we found that the distribution of intact TEs and fragmentary TEs is very different. On average, the former is more significantly closer to the gene than the latter (**Figure 2E**), meaning that intact TE is more likely to insert genes or gene regulatory regions, thereby affecting gene function (Niu et al., 2022), which a way of rapid evolution, and may also be one of the important ways for the *H. tibetana* genome to adapt to rapid variation habitats. Furthermore, as mentioned above, biochemical plasticity or gene expression is of extreme importance for high altitude organisms (Nicotra et al., 2010), and there are studies have shown that TEs can act as translators of phenotypic plasticity (Pimpinelli and Piacentini, 2020). But whether more TEs can result in higher plasticity is unclear. Thus, more TEs in the *H. tibetana* genome were both a cause of it adapting to extreme habitats and a consequence of it growing in extreme habitats.

From the perspective of a longer evolutionary history, the diploidization after tetraploidization is also a process through chromosome breakage, rearrangement and fusion, and studies have shown that these processes can change the expression and function of genes (Shi et al., 2015; Wang et al., 2022). These results suggest that the evolution of proteins is only a small part, and the genome responds more in other ways, including regulating the copy number of genes, changing regulatory patterns, etc.

## Data availability statement

The original contributions presented in the study are publicly available. This data can be found here: NCBI, PRJNA796061 and China National GeneBank Database, CNP0003543.

## Author contributions

LQ and ZW designed the research. RW, LQ and ZW collected the samples and performed the research. BW, JJ, ZS, TZ, and RW analyzed the data. RW wrote the paper. All authors read and approved the final manuscript.
