# Supplementary material for: How to survive in the world’s third poplar: Insights from the genome of the highest altitude woody plant, Hippophae tibetana (Elaeagnaceae)
Source: Front Plant Sci. 2022 Dec 14;13:1051587. doi: 10.3389/fpls.2022.1051587 (PMC9797102; doi:10.3389/fpls.2022.1051587)
Supplement: Supplementary file 1 [file DataSheet_1.pdf]

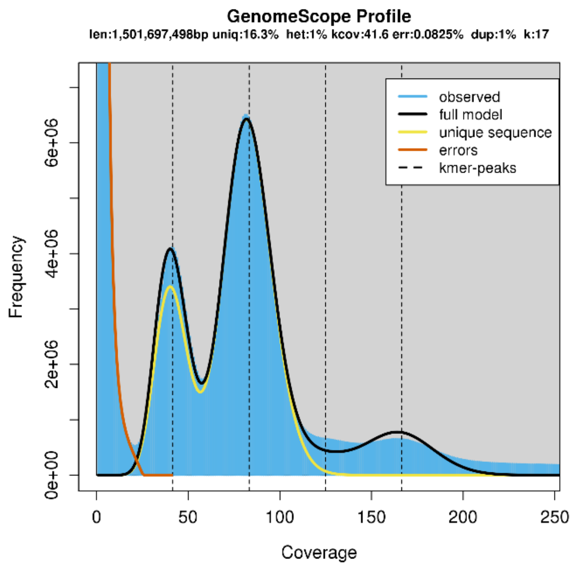

Figure S1. **Genome size estimation by GenomeScope.** GenomeScope profile of the *H. tibetana* genome inferred using a k-mer size of 17. Heterozygosity was estimated to be 1%. The genome haploid length and the proportion of repetitive sequences were estimated as 1.43 Gb and 83.7%, respectively.

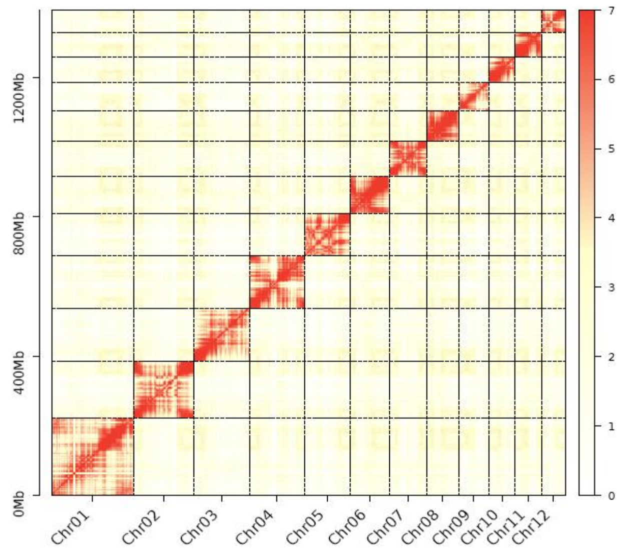

Figure S2. Interaction frequency distribution of Hi-C links among chromosomes. Genome-wide all-by-all Hi-C interaction matrix for sea-buckthorn chromosomes assembly using 12 clusters.

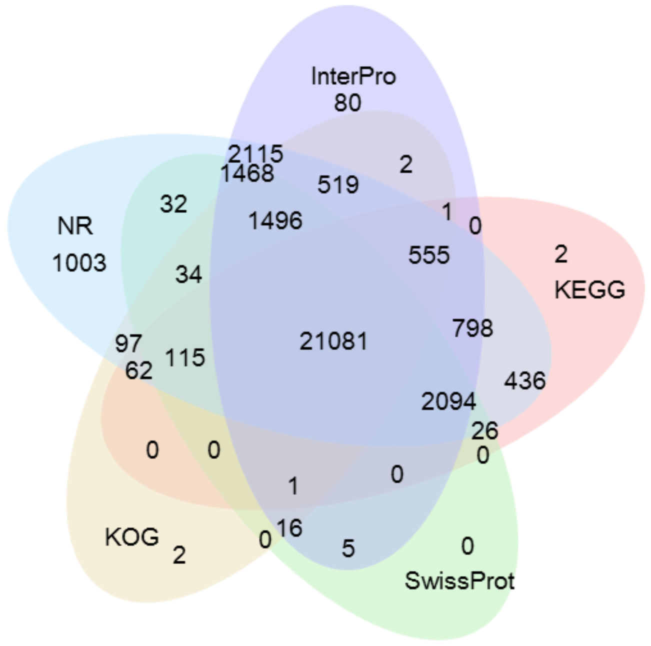

Figure S3. Functional annotation Venn diagram of *H. tibetana* protein coding genes using multiple protein related databases. Eukaryotic orthologous group (KOG), non-redundant protein database (NR), Kyoto encyclopedia of genes and genomes (KEGG), Swiss-Prot and InterPro.

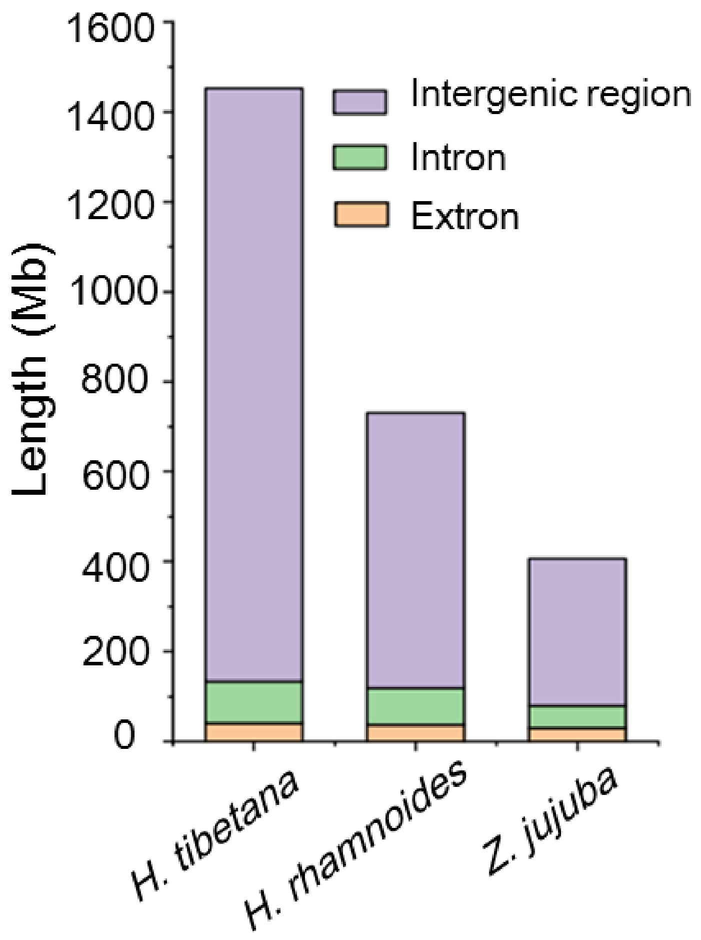

Figure S4. The proportions of extron, intron and intergenic region in *H. tibetana* genome.

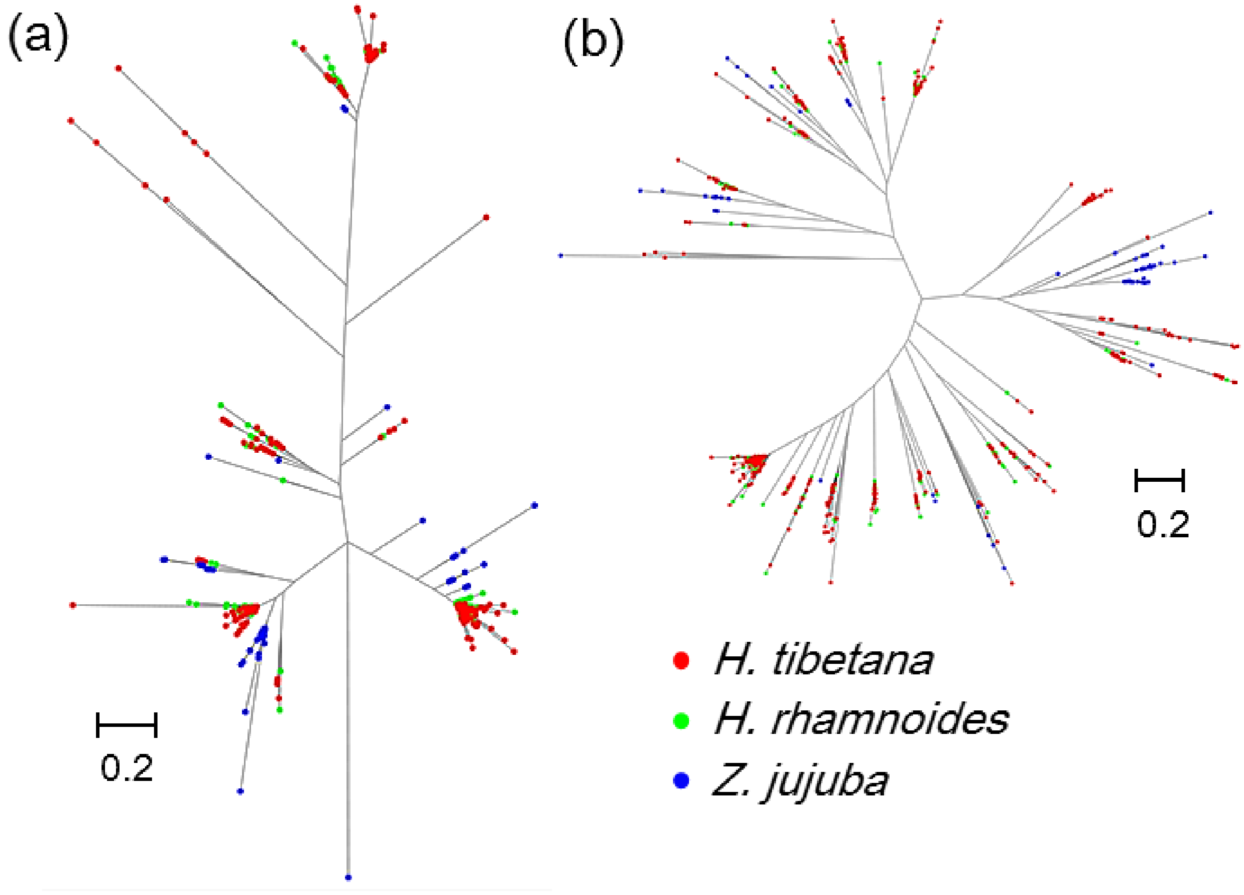

Figure S5. Phylogeny reconstruction of (a) Ty3/Gypsy type and (b) Ty1/Copia type LTR retrotransposons.

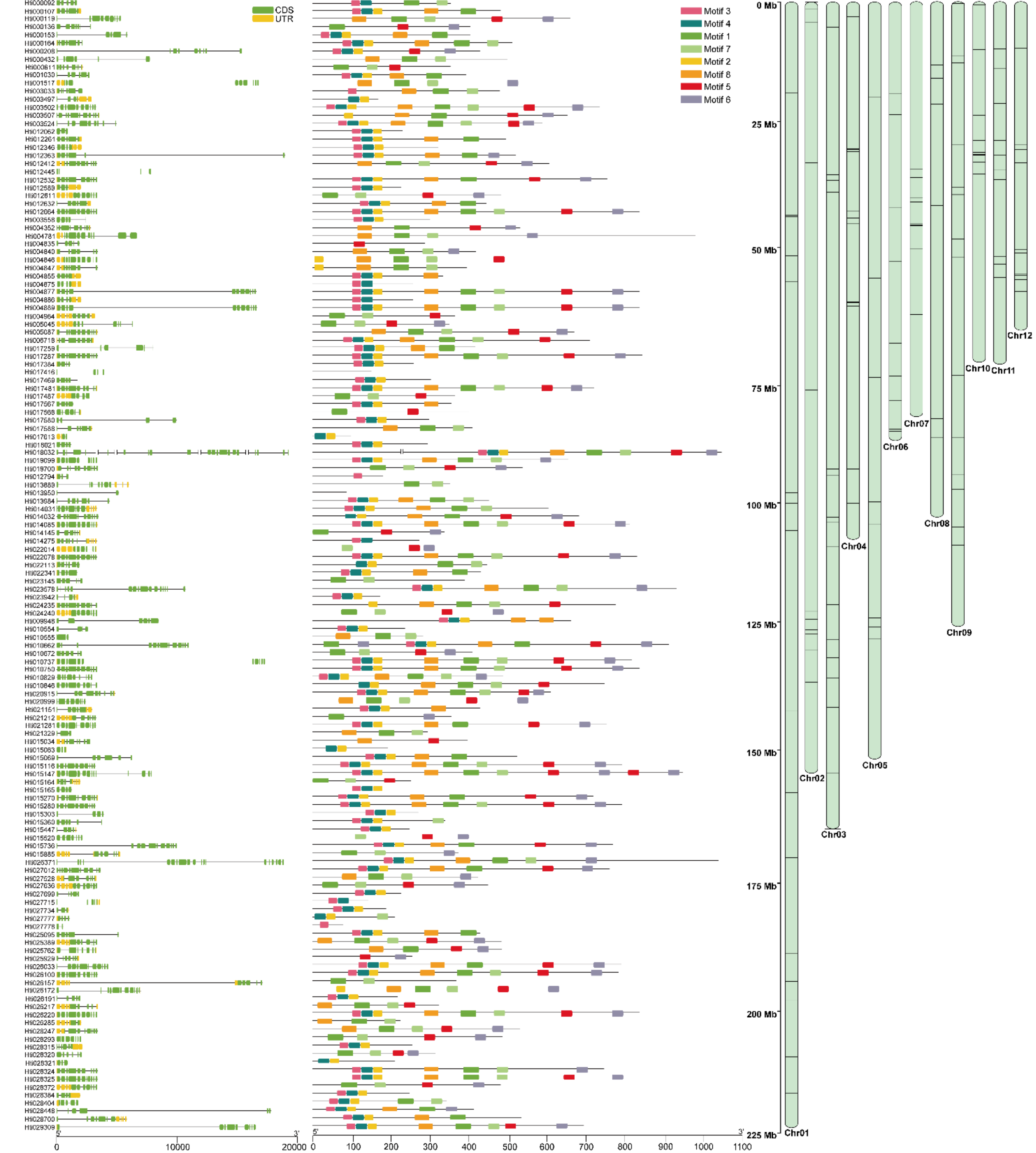

Figure S6. The distribution and structural characteristics of OG42 DNA transposons.

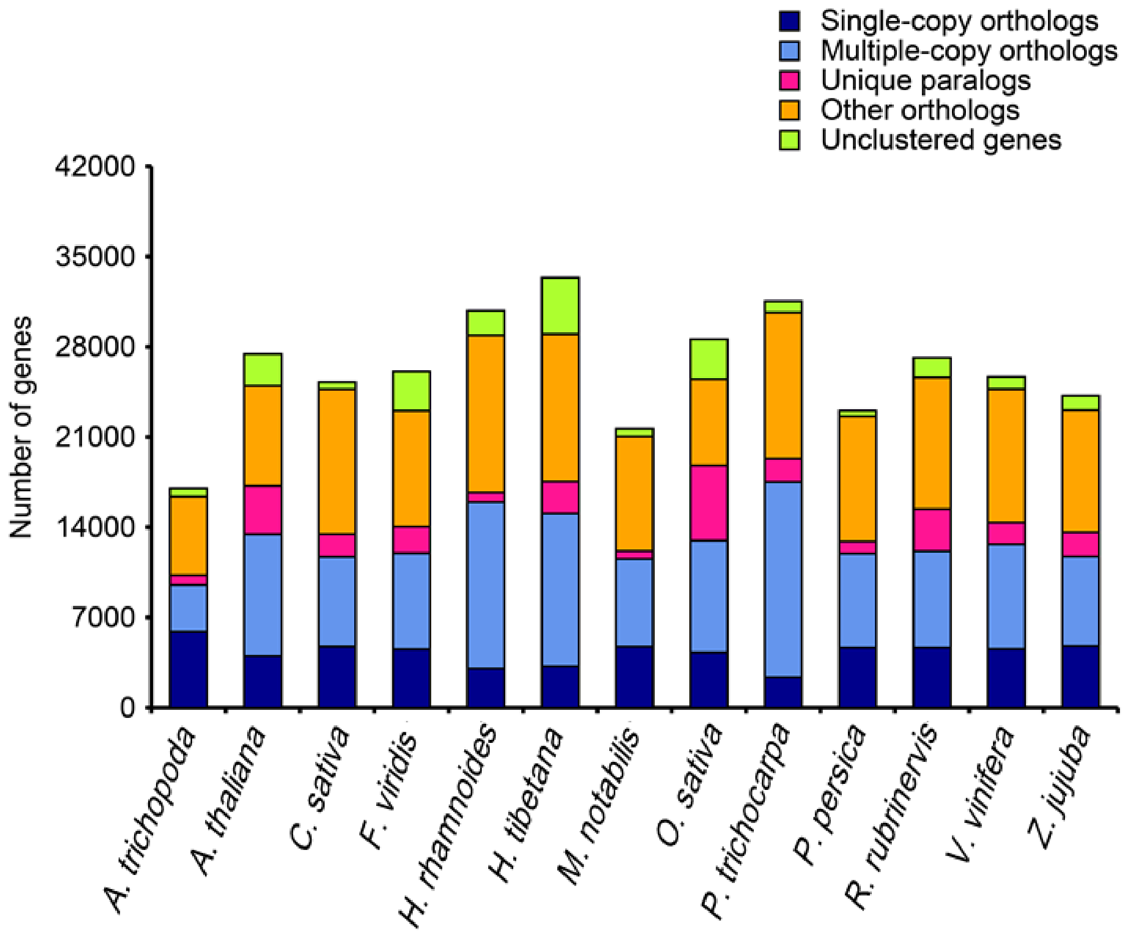

Figure S7. Comparison of gene families among *H. tibetana* and other 12 species. Clusters of orthologous and paralogous gene families in *H. tibetana* and 12 more fully sequenced plant genomes. Only the longest isoform for each gene was used. Gene families were identified using the OrthoMCL package with the default parameters.

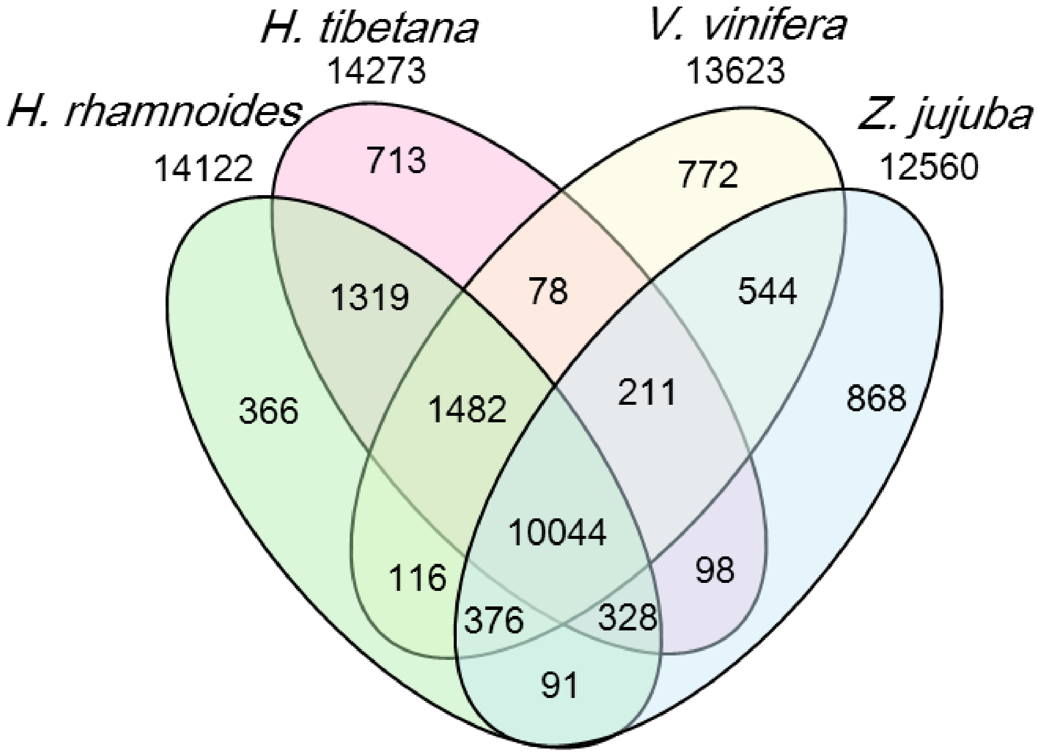

Figure S8. Venn diagram of shared and specific gene family numbers among *Hippophae tibetana* and three other species (*Ziziphus jujuba, Vitis vinifera* and *Hippophae rhamnoides*).

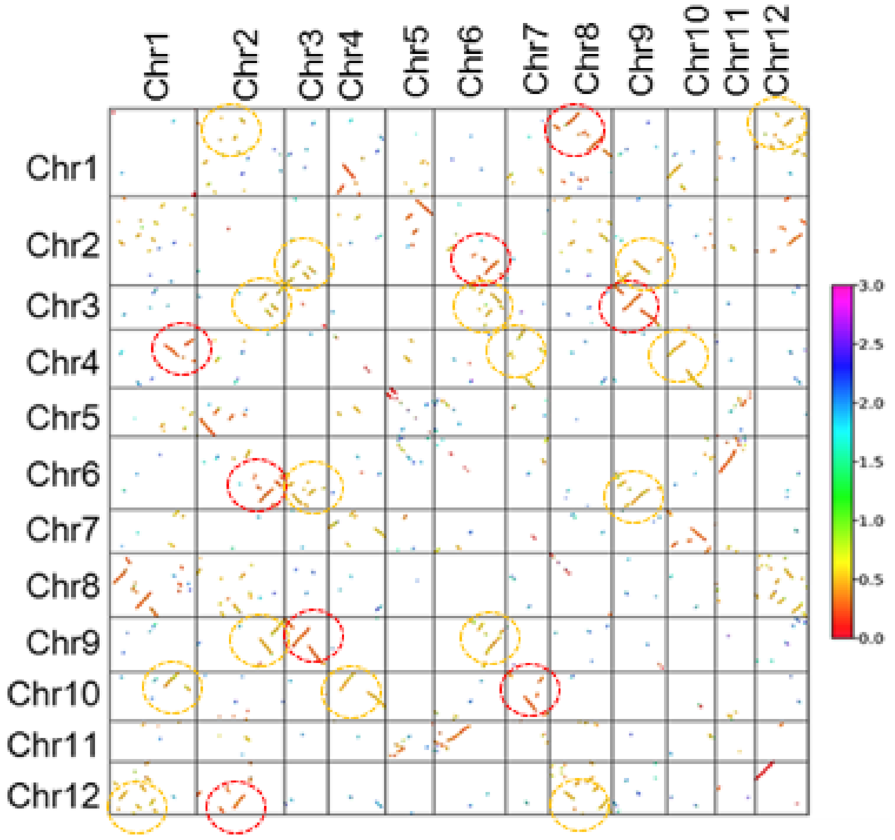

Figure S9. Dot plots of paralogs identified across Contigs in the *H. tibetana* genome. Dotted circle highlight examples of significant duplicate events.

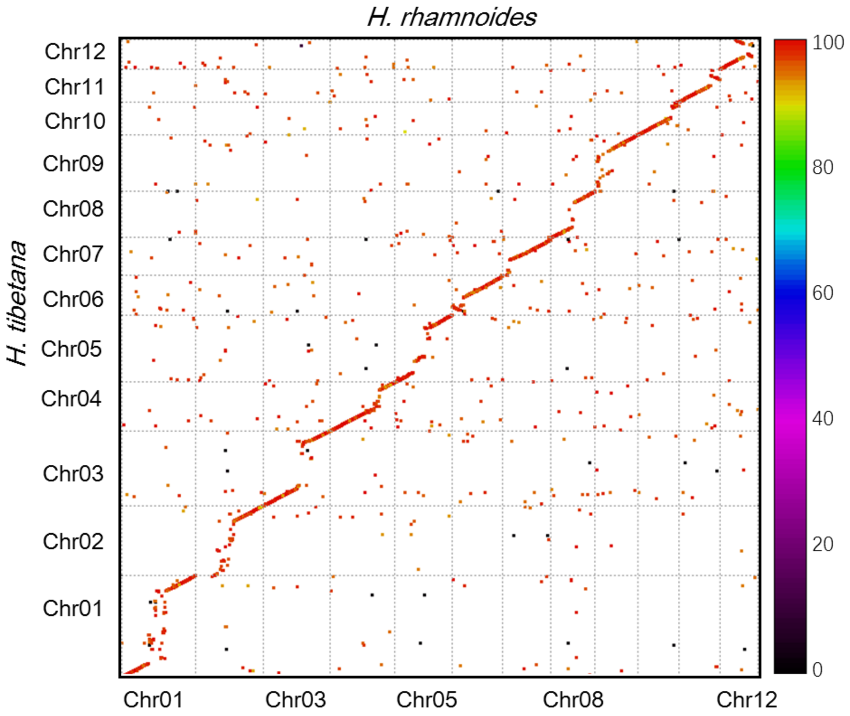

Figure S10. Genomic collinearity analysis of *H. tibetana* and *H. rhamnoides.*

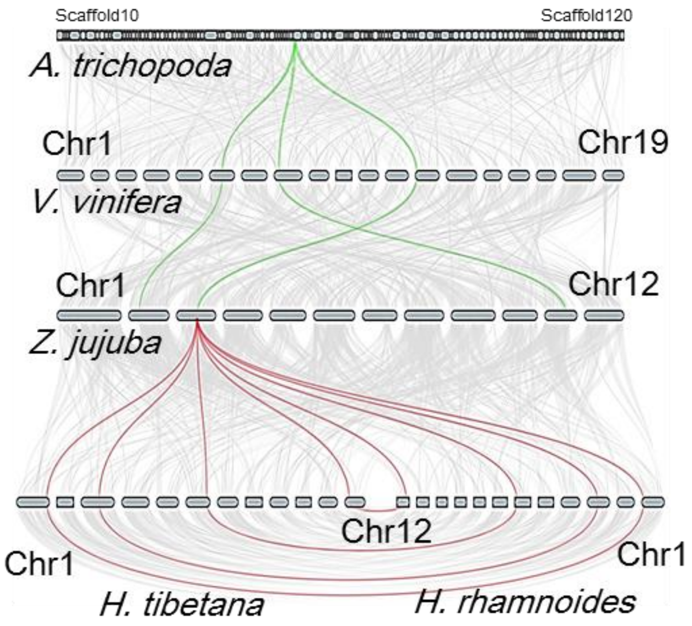

Figure S11. Syntenic gene blocks among *A. trichopoda, Z. jujuba, V. vinifera, H. rhamnoides and H. tibetana*. Examples of microsynteny connected by colored lines highlight the occurrence of two genome-wide duplications in seabuckthorn.

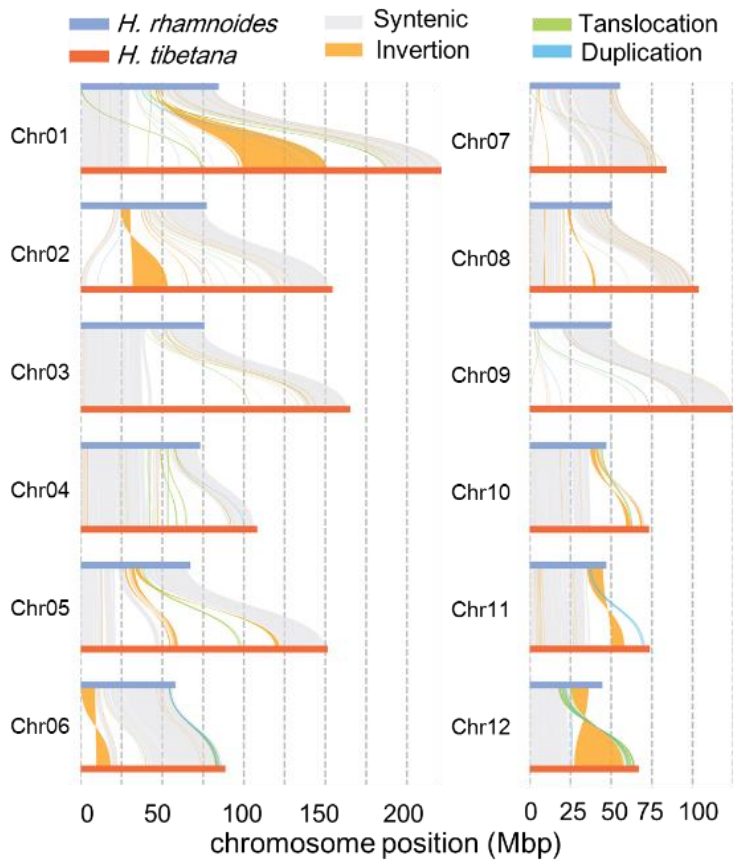

Figure S12. Syntenic blocks between the genomes of *H. rhamnoides* and *H. tibetana*. The syntenic alignment suggested high similarity between the karyotypes of two *Hippophae* species. Syntenic analysis was performed using McScan, and genes were visualized in blocks of 10.

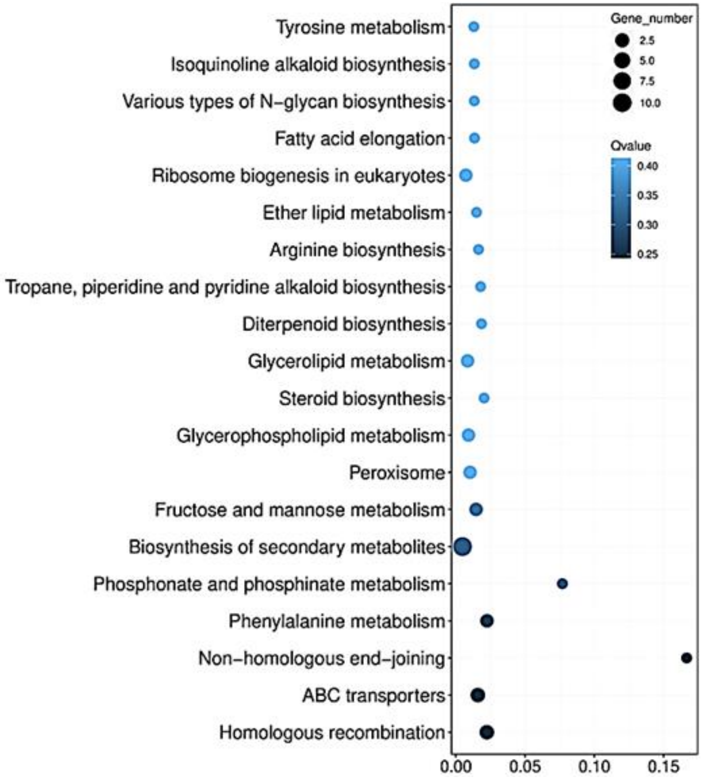

Figure S13. Functional enrichment of the positive selection genes. The gene number and the significance of each GO term were indicated by size and color of dot.

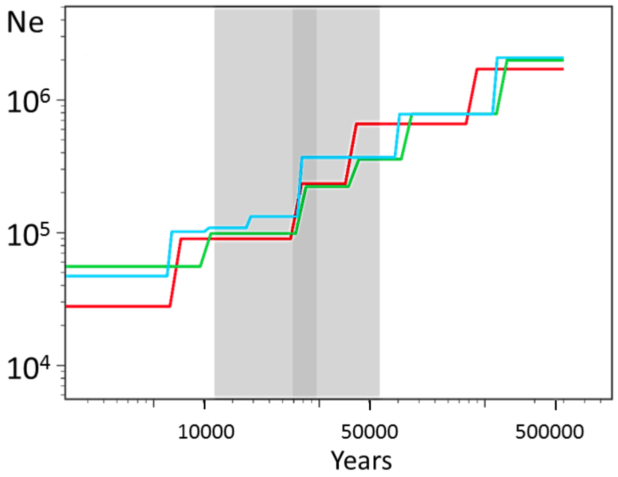

Figure S14. Demographic history of *H. tibetana* inferred with SMC++.
